# Assessment of the societal cost of *Taenia solium* in Angónia district, Mozambique

**DOI:** 10.1186/s12879-018-3030-z

**Published:** 2018-03-13

**Authors:** Chiara Trevisan, Brecht Devleesschauwer, Nicolas Praet, Alberto Pondja, Yunus Amade Assane, Pierre Dorny, Stig Milan Thamsborg, Pascal Magnussen, Maria Vang Johansen

**Affiliations:** 1 Department of Veterinary and Animal Sciences, Faculty of Health and Medical Sciences, University of Copenhagen, Frederiksberg, Denmark; 2 Department of Biomedical Sciences, Institute of Tropical Medicine, Antwerp, Belgium; 3 Department of Public Health and Surveillance, Scientific Institute of Public Health, Brussels, Belgium; 4 Faculty of Veterinary Medicine, Eduardo Mondlane University, Maputo, Mozambique; 5 Department of Neurology, University of Pretoria, Pretoria, South Africa; 6 Medical Faculty, Eduardo Mondlane University, Maputo, Mozambique; 7 Department of Virology, Parasitology and Immunology, Faculty of Veterinary Medicine, Ghent University, Merelbeke, Belgium; 8 Centre for Medical Parasitology, Department of Immunology and Microbiology, Faculty of Health and Medical Sciences, University of Copenhagen, Copenhagen, Denmark

**Keywords:** *Taenia solium* taeniosis/cysticercosis, Neurocysticercosis, Epilepsy, Migraine, Tension-type headache, Disability-adjusted life years: Societal cost, Zoonoses

## Abstract

**Background:**

The zoonotic parasite *Taenia solium* is endemic in Angónia district, Tete province, Mozambique, though the burden of the disease complex is unknown.

**Methods:**

As part of two cross-sectional studies on human and porcine cysticercosis in the area, unique epidemiological and cost data were collected in Angónia district, Mozambique in 2007. These data provided the basis for the assessment of the societal cost of *T. solium* in the district, which estimates the impact of the disease on human and pig populations and includes both health and economic approaches in the analysis.

**Results:**

Approximately 0.7% (95% Uncertainty Interval (UI), 0.4–0.9) and 0.4% (95% UI, 0.2–0.6) of the total population in the district was estimated to suffer from neurocysticercosis (NCC)-associated epilepsy and headache. The estimated average number of disability-adjusted life years (DALYs) due to NCC-associated epilepsy and headache was 6 (95% UI, 4–8) per thousand persons per year. The total annual costs due to *T. solium* cysticercosis were estimated at 90,000 USD (95% UI, 39,483–201,463) of which 72% (95% UI, 45–91) were costs linked to human cysticercosis and 28% (95% UI, 9.5–55) to pig production losses. The annual economic burden per NCC-associated epilepsy case in the district amounted to 33 USD (95% UI, 10–76).

**Conclusions:**

In this highly endemic area of Mozambique a large number of individuals suffer from symptoms associated with NCC. Healthy years of life are lost and people are left living with disabilities. Infected pork poses a serious risk to the community and affects the economy of smallholder farmers. Cost for treatment and hospitalization of patients with NCC-associated epilepsy, and lack of productivity and inability of suffering patients to work, further hinder socioeconomic development. Feasible solutions framed within a country specific algorithm and stepwise approaches are needed to control the parasite in the country.

## Background

*Taenia solium* taeniosis/cysticercosis (TSTC) is a zoonotic disease complex currently challenging the public health and agricultural sectors in many low-income countries. *Taenia solium* is endemic in countries of Africa, Asia and Latin America, hindering growth and socioeconomic development [1–3].

Humans acquire the adult tapeworm (taeniosis) by eating raw or undercooked pork containing cysticerci. Cysticercosis is contracted by both humans and pigs after accidentally ingesting *T. solium* eggs in faeces from humans harbouring the adult tapeworm. In humans cysticerci may lodge in the central nervous system leading to neurocysticercosis (NCC). The disease may cause severe disorders, the most common being epilepsy and headache [4], though people can also remain asymptomatic [5].

Recent findings reported that seizures also occur in pigs with NCC and disease negatively affects their behaviour, posing a further burden on the welfare of affected animals [6, 7].

In 2013, the World Health Assembly passed the WHA66.12 resolution with the aim of eliminating TSTC as a public health problem [8]. Recent results of a study coordinated by the Foodborne Disease Burden Epidemiology Reference Group ranked *T. solium* as the most important foodborne parasite in the world causing a great number NCC-associated epilepsy cases and deaths, resulting in 2.8 million disability-adjusted life years (DALYs) [9]. In parallel to these studies, country-specific studies have been conducted to estimate the health and economic burden of TSTC [10–14]. These highlighted the lack of representative country-specific data and the necessity of making assumptions, possibly leading to under- or overestimations of the impact of the parasite on human and pig populations.

During the past decade studies on *T. solium* were conducted in Angónia district and results have shown that the parasite and all conditions for its transmission are present [15, 16]. The parasite is likely to pose a large burden to smallholder farmers and their society in this area of the country. Angónia district is part of Tete province, located in north-western Mozambique. Smallholder farmers living in close relation to their animals mostly populate the area. Pig production plays a major role in the families’ economic activity in the district [17]. In recent years the number of pigs per family has increased as farmers have seen pigs to have a quick economic turnover and are easily raised as they can be left to roam freely. However, the economic growth and development of the area has been hindered by many post-civil war socio-economic and political challenges [18]. Political instability has led to the regress of the district, making it one of the poorest of the country [19]. Lack of access to health care, education, water supply and sanitation constantly exacerbate the poor living conditions of smallholder farmers and their families.

The present study aimed at quantifying the societal cost of *T. solium* in Angónia district by estimating the impact of the disease on human and pig populations and including both health and economic approaches in the analysis.

## Methods

### Study area and population

In 2007 the total human population of the district was 330,328 people. The main occupation and most important economic activity of the district was mixed farming, including pig rearing. Pigs were mostly free-roaming during the day and dry seasons and kept confined at night and during the rainy, crop growing season [17, 20].

Health care access was limited in the district with only one rural hospital, four health centres and three health points and inhabitants commonly consulted traditional healers. No computed tomography (CT) scanner was available [17].

Necessary conditions for transmission of the parasite were present, namely lack of knowledge about *T. solium*, open defecation, poor sanitation, free roaming pigs, lack of slaughtering facilities with qualified meat inspectors, home slaughtering and consumption of uninspected and undercooked pork [15, 20].

Between September and November 2007 two cross-sectional studies on human and porcine cysticercosis, respectively, were conducted in Angónia district, Mozambique. The lifetime prevalence of epilepsy, where screen-positive individuals were reviewed and confirmed by a neurologist, was estimated including 1723 study subjects in the study and the results on the proportion of patients with epilepsy suffering from NCC were reported according to Del Brutto et al. (2001) [21] criteria [15]. Proportions of severe progressive headache (migraine and tension-type) among patients with epilepsy were based on answers provided by structured interviews from questionnaire developed by the Cysticercosis Working Group in Eastern and Southern Africa (CWGESA) [22, 23]. Pondja et al. (2010) [20] conducted a study to estimate the prevalence of porcine cysticercosis including 661 pigs from 306 households in 11 villages across the district. The B158/B60 ELISA detecting circulating cysticercus antigens and tongue examination for submucosal cysticerci were used to diagnose porcine cysticercosis.

### Health burden assessment

The health burden for the year 2007 was assessed by estimating the annual number of NCC-associated epilepsy, migraine and tension-type headache cases, and the resulting number of deaths and DALYs. Table 1 presents the parameters and related distributions used in the analysis.

**Table 1:** Parameters used to estimate the health burden due to NCC^a^-associated epilepsy and headache (migraine and tension-type) in Angónia district

Parameter	Mean (95% UI^b^)	Distributions	Reference
Mortality due to epilepsy per 1000	0.08 (0.076–0.084)	Uniform (7.6e-05, 8.4e-05)	[28]
Prevalence of active epilepsy (%)	1.3 (0.8–1.9)	Beta (22, 1701)	[15]
*Proportions*			
Epilepsy associated to NCC (%)	51 (43–59)	Uniform (0.427, 0.592)	[15]
Epilepsy patients receiving treatment (%)	15 (9.4–21)	Beta (22, 129)	[15]
Headache among people with lifetime epilepsy (%)	62 (54–70)	Beta (94, 57)	[23]
Migraine among people with headache and epilepsy (%)	39 (30–50)	Beta (37, 57)	[23]
Tension-type headache among people with headache and epilepsy (%)	61 (50–70)	Beta (57, 37)	[23]
*Disability weights*			
Epilepsy			GBD^c^ 2010 [31]
Untreated	0.426 (0.286–0.565)	Uniform (0.279, 0.572)	
Treated, seizure free	0.076 (0.048–0.105)	Uniform (0.047, 0.106)	
Headache			GBD^c^ 2013 [32]
Migraine	0.441 (0.301–0.581)	Uniform (0.294, 0.588)	
Tension-type	0.040 (0.023–0.056)	Uniform (0.022, 0.057)	

^a^ *NCC* neurocysticercosis; ^b^ *UI* uncertainty interval; ^c^ GBD 2010, 2013: Global Burden of Disease Study 2010 and 2013

Epilepsy was defined as two or more unprovoked seizures unrelated to any acute intracranial disease (e.g. cerebral malaria, meningitis), acute metabolic disorders, withdrawal of drugs or use of alcohol and occurring at least 24 h apart [24]. Lifetime prevalence of epilepsy was defined as the proportion of patients identified with a history of epilepsy at any time, regardless of treatment or recent seizure activity, while active epilepsy was defined as having had at least one seizure in the past year and receiving or having received treatment [25]. The prevalence of active epilepsy was 1.3% (95% Uncertainty interval (UI), 0.8–2.0), obtained by dividing the number of lifetime epilepsy patients receiving epilepsy treatment by the total number of study participants [23].

Patients with NCC might experience headaches that can present as migraine or tension-type headache [26, 27]. Periods of recurrent headaches were reported by 94 of 151 patients with lifetime epilepsy. Of these 94 patients had migraine and the rest had tension-type headache (Table 1).

**Fig. 1:**
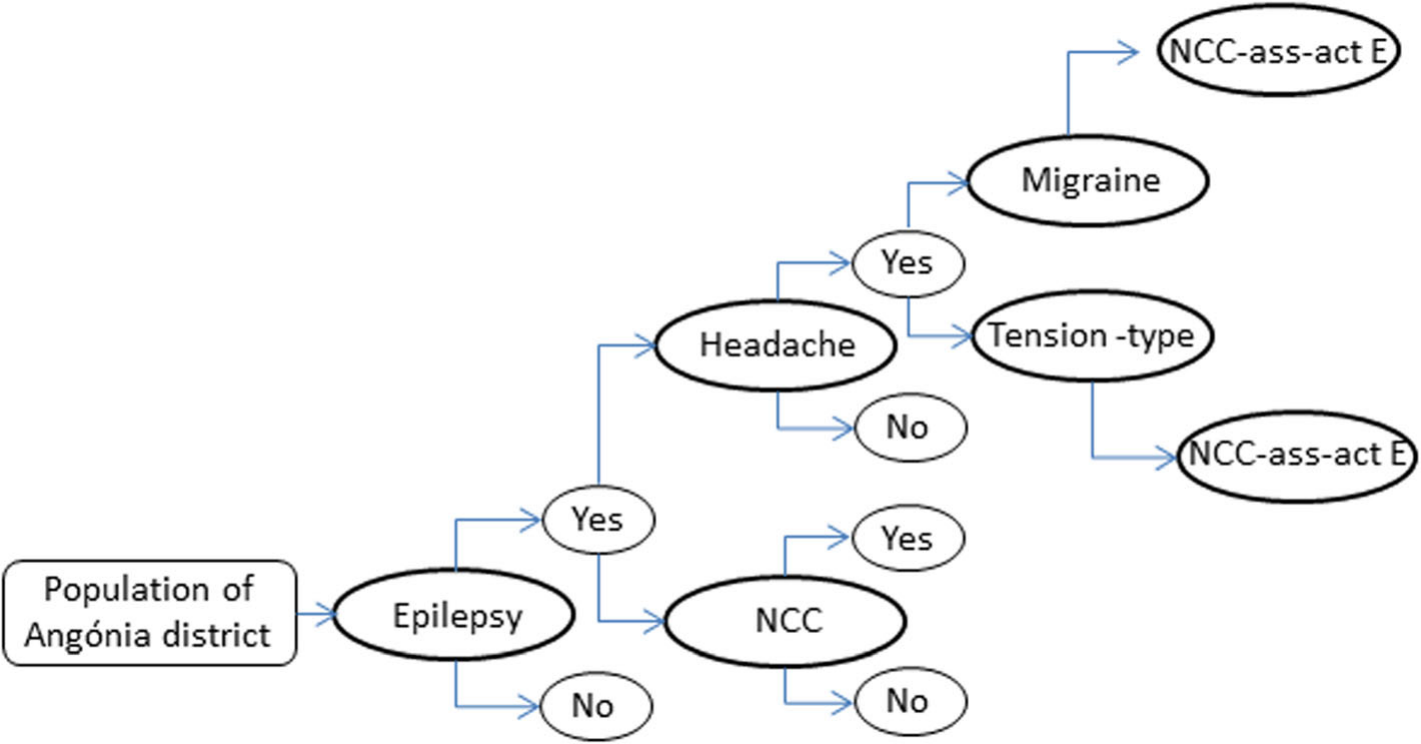
Health outcome tree to estimate the proportion of patients with NCC-associated epilepsy, migraine and tension-type headache in Angónia district, Mozambique

The burden was estimated among people with NCC-associated epilepsy, and headache was added as an extra burden on a subset of patients identified having NCC-associated epilepsy. A health outcome tree (Fig. 1) was used to obtain the proportions of the population with epilepsy, migraine and tension-type headache due to NCC.

To estimate the mortality due to NCC-associated epilepsy, the WHO mortality rate (2008) for epilepsy in Mozambique was multiplied by the proportion of NCC-associated active epilepsy in Angónia district [15, 28]. Data on mortality due to headache could not be retrieved, hence it was assumed that mortality due to migraine and tension-type headache would not occur.

The number of DALYs was estimated using known methods [29, 30]. In short: DALYs were obtained by summing years lived with disability (YLD) and years of life lost (YLL). The formulas used for the DALY calculation are described in eqs. 1 and 2.

1 $$ \mathrm{YLD}={\mathrm{P}}^{\ast }\ \mathrm{DW} $$

Where ‘P’ represents the number of prevalent cases and ‘DW’ is the disability weight.

2 $$ \mathrm{YLL}={\mathrm{N}}^{\ast }\ \mathrm{L} $$

Where ‘N’ is the number of deaths caused by disease per year and ‘L’ is the life expectancy at the age of death in years.

Disability weights for epilepsy, migraine and tension-type headache were based on those of the GBD study 2010 and 2013 [31, 32]. For life expectancy parameters, the GBD 2010 life expectancy table was used where life expectancy at birth is 86 years [33].

Results were computed using two variants of DALYs[*K*;*r*] (with *K* the age weighting constant and *r* the discount rate) and using a prevalence approach. The base case DALYs[0;0] were estimated without age weighting or time discounting, in line with current practice.

### Economic burden assessment

The epidemiological and economic parameters used for the economic assessment are summarised in Tables 2 and 3.

**Table 2:** Parameters used to estimate the economic burden of *T. solium* in Angónia district, Mozambique

Parameter	Mean (95% UI^a^)	Distribution	Reference
Study zone population	330,328	Fixed	[17]
Active epilepsy (%)	1.3 (0.8–2.0)	Beta (22, 1701)	[15]
NCC-associated epilepsy (%)	51 (43–59)	Uniform (0.427, 0.592)	[15]
Epilepsy patients consulting a traditional healer (%)	23 (18–28)	Multinomial (0.227)	[23]
Epilepsy patients consulting a physician, a nurse or neurologist (%)	9.1 (6–13.4)	Multinomial (0.091)	[23]
Epilepsy patients without treatment (%)	68 (62–74)	Multinomial (0.682)	[23]
Visits to a traditional healer in case of epilepsy (average times per year)	5.5 (1.2–9.8)	Uniform (1, 10)	[23]
Visits to a doctor in case of epilepsy (average times per year)	4.5 (1.2–7.8)	Uniform (1, 8)	[23]
Epilepsy patients with prescribed phenobarbital (%)	44 (33–55)	Beta (36, 46)	[23]
Epilepsy patients with injury referred to the hospital (%)	15 (11–20)	Beta (38, 222)	[23]
Length of stay in a hospital (average days per year)	11 (1.5–20)	Uniform (1, 21)	[23]
Loss of working time due to epilepsy (days per year)	16 (1.7–29)	Uniform (1, 30)	[23]
Unemployed due to epilepsy (%)	2.2 (0.9–5.1)	Beta (6, 261)	[23]
Working days per year	266 (222–310)	Uniform (220, 312)	Assumption
% of the population			
Economically active	40	Fixed	[17]
Not economically active	47	Fixed	[17]
Unemployed	14	Fixed	[17]
Pig population in the study area	20,411	Fixed	Assumption
Proportion of adult pigs sold per year (%)	33	Fixed	Assumption
Porcine cysticercosis prevalence based on tongue examination (%)	13 (10–16)	Beta (84, 577)	[20]

^a^ *UI* Uncertainty interval

**Table 3:** Cost parameters in USD used to estimate the economic burden of *T. solium* in Angónia district, Mozambique

Parameter	Mean (95% UI^a^)	Distribution	Reference
Average monthly salary	104 (30–178)	Gamma (5.3, 0.06)	[43]
Cost of a visit to a physician (public hospital)	3.1 (2.1–4.1)	Gamma (33.4, 11.1)	[44]
Cost of a traditional healer	63 (25–101)	Gamma (8.5, 0.15)	[23]
Cost of one day at the hospital	2.3	Fixed	[44]
Antiepileptic drugs (2 weeks treatment)	5	Fixed	[23]
Value of an adult pig	55 (30–79)	Gamma (16.5, 0.32)	(Gule, personal communication)
Value reduction of infected pork (%)	50	Fixed	(Gule, personal communication)

^a^ UI Uncertainty interval

Direct and indirect costs related to both human and porcine cysticercosis were included in the assessment. Direct costs related to human health included expenses for treatment such as: medical care, medicine and hospitalisation for patients with NCC-associated epilepsy. The price of a visit to a traditional healer in Angónia district varied from visit to visit hence an average price was calculated.

The indirect costs or productivity losses included the expenses related to individuals who were unable to go to work or unemployed because of NCC-associated epilepsy.

The number of working days per year was estimated at a minimum of 220 and a maximum of 312 days in order to take differences between the formal and informal sector into account.

**Fig. 2:**
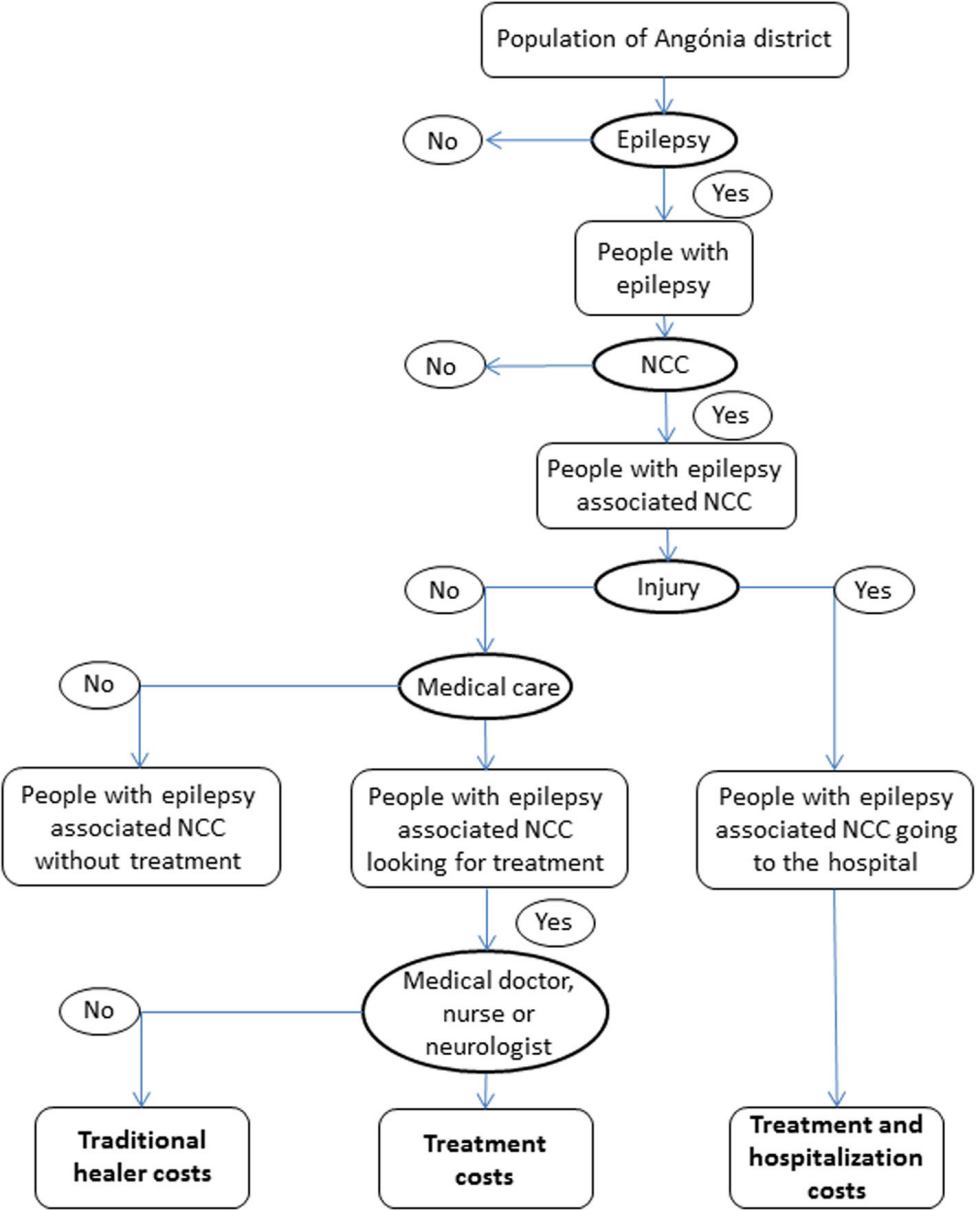
Decision tree to estimate the economic burden of NCC-associated epilepsy in Angónia district, Mozambique

A decision tree (Fig. 2) was used to obtain the proportions of the population with epilepsy due to NCC, with or without injury, treatment and hospitalization. The patients without injury were divided into those who sought medical care and those who did not. The patients with NCC-associated epilepsy that sought medical care were divided into: patients consulting a traditional healer and patients consulting a medical doctor, nurse or neurologist. It was assumed that all patients that got injured during an epileptic seizure would refer to the hospital and be hospitalized and the ones without injury, that sought medical care, would be treated as out-patients.

For the estimation of the losses due to porcine cysticercosis, the cysticercosis prevalence based on tongue examination, 12.7% (95% UI, 10.3–15.5), was used. The prevalence was estimated on a sample of 661 pigs from Angónia district [20]. In Angónia district it was common practice for farmers to sell the pigs to pig traders who at purchase, would inspect the pig’s tongue for cysticercosis and agree on the price. The price of a pig depended on presence or absence of infection and the animal’s size. In the region, the average price reduction of a cysticercosis infected pig at slaughter was of 50% (Gule, personal communication). This was the only agricultural factor taken into account, as porcine cysticercosis has so far not shown to have an effect on animal’s productivity [4]. Losses due to carcass condemnation were not taken into account. In Angónia district meat inspection was present only at one market place, therefore the likelihood of infected meat reaching market there was very low [34]. Because reliable information on the number of slaughtering pigs was not available, the slaughtering pig population was estimated at 20,411 pigs. This number was based on the assumption that a smallholder pig farmer in Angónia district on average slaughtered one pig annually, and pigs were present in every fourth household [17]. We further assumed that only one third of the slaughtering pigs would be sold per year, as pig production for personal consumption is common in the area [20].

### Health and economic burden assessment analyses

All analyses were performed in R 3.2.2 [35]. Scripts are available online at https://github.com/brechtdv/tsol-mozambique. The uncertainty of the parameters was modelled using Monte Carlo simulations. This allowed calculating 95% uncertainty intervals (UI) for each result. Different distributions were used according to the type of information available for each of the variables in Tables 1, 2 and 3. The number of iterations was set to 100,000. Sensitivity analyses were conducted to show the contribution of each uncertain variable to the overall uncertainty of the end result. The impact of the uncertainty in the different parameters on the overall uncertainty in total DALYs, costs and potential losses is shown by the partial correlation coefficients presented in the results section.

## Results

### Health burden assessment

Approximately 0.7% (95% UI, 0.4–0.9) and 0.4% (95% UI, 0.2–0.6) of the total population in the district was estimated to suffer from NCC-associated epilepsy and headache, respectively. Around 85% (95% UI, 79–91) of the diseased population never received any treatment (Table 4). Overall, it was estimated that NCC-associated epilepsy, migraine and tension-type headache led to more than 2003 (95% UI, 1433–2762) healthy life years lost (Table 5), meaning 6 (95% UI, 4–8) DALYs per 1000 persons per year. Non-fatal health outcomes were found to be the largest contributors to the overall health impact (53% (95% UI, 39–65). YLDs associated with NCC-headache contributed to 13% (95% UI, 8–19) of the total DALY estimate.

**Table 4:** Estimated number of people with NCC-associated epilepsy, migraine and tension-type headache, people suffering without treatment, people dying due to NCC-associated epilepsy and pigs with cysticercosis in Angónia district, Mozambique

Estimate	Mean (95% UI^a^)	% of total population (95% UI^a^)
Annual number of prevalent cases of NCC-associated epilepsy	2151 (1299–3249)	0.7 (0.4–0.9)
People with NCC-associated epilepsy without treatment	1837 (1103–2788)	0.6 (0.3–0.8)
Number of deaths due to NCC-associated epilepsy	14 (11–16)	0.004 (0.003–0.005)
Annual number of prevalent cases of NCC-associated migraine	527 (290–855)	0.2 (0.1–0.3)
Annual number of prevalent cases of NCC-associated tension-type headache	812 (468–1274)	0.2 (0.1–0.4)
Number of pigs with cysticercosis	2595 (2098–3133)	12.7 (10.3–15.3)

^a^ UI Uncertainty interval

**Table 5:** Estimated prevalent Years Lived with Disability (YLDs), Years of Life Lost (YLLs) and Disability-Adjusted Life Years (DALYs) for NCC-associated epilepsy, migraine and tension-type headache in Angónia district, Mozambique

Estimate	Mean (95% UI^a^)	% contribution to total DALY (95% UI^a^)
YLD NCC-associated epilepsy	806 (415–1368)	39 (28–51)
YLD NCC-associated migraine	233 (111–415)	11 (7–18)
YLD NCC-associated tension-type headache	32 (14–59)	1.6 (0.8–2.6)
YLL NCC-associated epilepsy	932 (781–1088)	47 (35–61)
Total DALYs*	2003 (1433–2762)	100
DALYs per 1000 persons	6 (4–8)	100

^a^ UI Uncertainty interval

When using non-uniform age weighting and a 3% discount rate, the estimated number of DALYs[1;0.03] was 1618 (95% UI, 1042–2411) and the estimated number of DALYs[1;0.03] per thousand person per year was 4.9 (95% UI, 3.2–7.2). Of the total DALYs[1;0.03], 73% (95% UI, 61–82) were attributed to YLD and 27% (95% UI, 18–39) to YLL, respectively.

### Economic burden assessment

*Taenia solium* led to an economic loss of around 90,000 USD (95% UI, 39,483–201,463) of which 72% (95% UI, 45–91) were costs linked to human cysticercosis and 28% (95% UI, 9.5–55) were due to pig production losses (Table 6). The annual economic burden per case of NCC-associated epilepsy in the district amounted at 33 USD (95% UI, 10–76). The economic burden of NCC-associated epilepsy was dominated in particular by the production losses. Direct costs due to hospitalization were the next major contributor to the losses, although contributed only to 12% (95% UI, 5–24) of the total costs for NCC-associated epilepsy.

**Table 6:** Estimated direct and indirect annual costs due to *T. solium* in humans and pigs in Angónia district, Mozambique

Type of cost	Mean USD	(95% UI^a^)
Hospital	7945	(4312–13,019)
Antiepileptic treatment	1501	(1038–2100)
Medical doctor (public hospital)	14	(3–27)
Traditional healer	312	(56–758)
Inactivity	61,462	(11,131–166,964)
Pig losses	22,282	(12,315–35,647)
Total costs	93,370	(39,483–201,463)
Price per NCC-associated epilepsy case	33	(10–76)

^a^ *UI* Uncertainty interva

Agricultural losses due to reduced value of slaughtered pigs accounted for almost one third of the total costs (28% (95% UI, 9.5–55)).

### Sensitivity analyses

**Fig. 3:**
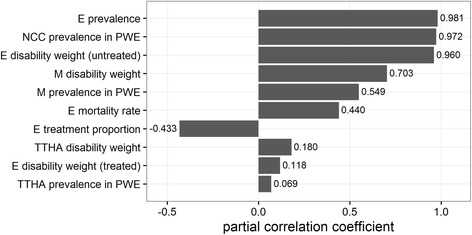
Partial correlation coefficients showing which parameter influences the estimated DALYs due to NCC-associated epilepsy, migraine and tension-type headache for the year 2007 in Angónia district, Mozambique. E-epilepsy; NCC-neurocysticercosis; PWE-people with epilepsy; M-migraine; TTHA-tension-type headache

**Fig. 4:**
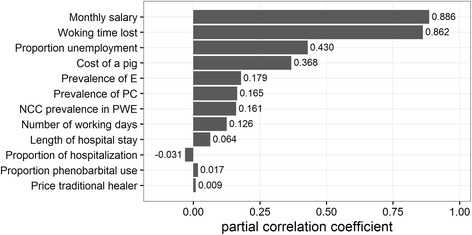
Partial correlation coefficients showing which parameter influences the estimated economic burden due to NCC-associated epilepsy and porcine cysticercosis for the year 2007 in Angónia district, Mozambique. E-epilepsy; PC-porcine cysticercosis NCC-neurocysticercosis; PWE-people with epilepsy

Figures 3 and 4 show the partial correlations coefficients, displaying the impact of the different parameters on the uncertainty of the overall estimates.

## Discussion

The study has shown that in this *T. solium* endemic area of Mozambique the parasite affects the livelihood of subsistence farmers and their society by reducing their societal and economic wellbeing. Based on data, collected in parallel from human and pigs in Angónia district, the present assessment revealed that NCC-associated epilepsy and headache led to 6 (95% UI, 4–8) DALYs per thousand persons per year, costing this society more than 90,000 (95% UI, 39,483–201,463) USD. Compared to other studies assessing the burden of *T. solium,* the estimated DALYs of this study were higher whereas the cost estimates for the district were lower.

This study is the third to use DALYs to estimate the health burden of *T. solium* in Africa and the second to include headache data in the assessment. In Africa, the first, carried out in Cameroon, estimated a number of 9.0 DALYs per 1000 person-years [13], while the second, carried out in Tanzania, estimated a number of 0.7 DALYs per 1000 person-years [14]. Health burden assessments have also been performed in Mexico, Nepal and India, where the author estimated 0.25 (0.12–0.46), 0.5 (0.2–1.1) and 1.7 (0.8–3.4) DALYs per thousand persons per year, respectively [10, 12, 36]. The reason for the high health burden in Angónia district is likely to be an added effect of a) being a rural area with low socio-economic status, b) the presence of all risk factors for *T. solium* and c) no awareness of the disease in the health or agricultural sectors. In Angónia district, the prevalence of epilepsy was twice as high and the proportion of NCC in subjects with epilepsy was three times as high compared to Tanzania. To estimate the years of life lost we used the GBD 2010 life expectancy, these increased the results1.25 fold compared to using the standard life expectancy table of Coale-Demeny [37] and finally the inclusion of headache further increased the result 1.1 fold.

In Angónia district around 85% (95% UI, 79–91) of the patients with NCC-associated epilepsy never received treatment while in Tanzania over half of the diseased population was left untreated. As the disability weights for untreated epilepsy cases are higher than the ones for treated epilepsy, a higher number of DALYs per 1000 persons per year was expected in the district.

Headache data were included for the first time in an assessment of the burden of *T. solium* in Africa. Based on the data, the model estimated that in Angónia district 0.4% (95% UI, 0.2–0.6) of the total population suffered from NCC-associated headache. Bhattarai et al. (2012) assessed the burden of NCC-associated headache in Mexico and estimated that 0.08% of the population was suffering. The results in the current study are five times higher. In this study, headache was added as an additional burden on a subset of patients with NCC-associated epilepsy, partly explaining a higher burden in Angónia district. To estimate the burden of NCC-associated headache in Mexico, Bhattarai et al. (2012) used the proportion of NCC-associated headache found in a systematic review by Carabin et al. (2011). As the review did not mention what type of headache was taken into consideration, we preferred not to use such extrapolation but use a different approach. In this study migraine and tension-type headache were added as an additional burden for a subset of patients with NCC-associated epilepsy, however NCC associated headache in non-epileptics was not calculated.

In our assessments the contribution of YLD and YLL to the total DALY was 53% and 47%, respectively. These figures indicate that in this study the disease had almost an equal impact on the life quality and life loss of the inhabitants. However when using non-uniform age weighting and a 3% discount rate 73% (95% UI, 61–82) were attributed to YLD and 27% (95% UI, 18–39) to YLL, respectively, in contrast with results of the study in Cameroon, where the percentages of YLD and YLL were 15% and 85%, respectively.

The results of the cost estimation showed that *T. solium* contributes to a high economic impact in Angónia district. Around 90,000 USD were lost due to the disease annually. Per NCC-associated epilepsy case the annual economic burden in the district amounted at 33 USD (95% UI, 10–76). Compared to other studies that assessed the economic burden of *T. solium*, these results are low. In Tanzania and Cameroon, the cost per NCC-associated epilepsy case was more than three and seven times higher, respectively. The difference can partly be explained by the large treatment gap, and partly by the low treatment costs.

The losses due to inactivity were 82% of the costs for NCC-associated epilepsy. This percentage is in line with that in Cameroon, where 89% of the total costs due to NCC-associated epilepsy were lost due to inactivity and Tanzania where around half the total costs were due to unemployment and/or inactivity of patients with NCC-associated epilepsy.

The losses due to porcine cysticercosis were estimated to be around 22,000 USD (0.07 USD per capita per infected pigs). This result is in line with estimates from Cameroon, where two studies found that 0.10 Euro per person per infected pig were lost [13, 38]. In Eastern Cape Province, South Africa the annual loss per person per infected pig was 0.60 Euro. The differences in losses might be reflected by the disease prevalence, the price reduction due to infection and pig value. The higher losses in Angónia district and in South Africa may be due to the higher prevalence of porcine cysticercosis observed in these two areas and to a different price reduction used in the estimations. In Angónia district the average value of an adult pig was 55 USD, while in Cameroon it was almost the double (100 Euro). Furthermore, in Angónia district the price reduction of a pig diagnosed with cysticercosis was 50%, while in Cameroon 30% (Gule, Personal communication) [13].

Angónia district was characterized by Pondja et al. (2010) as a rural area with very high level of illiteracy, free roaming pigs, lack of proper slaughtering facilities, frequent consumption of undercooked, uninspected pork, open latrines allowing pigs’ free access, and no awareness of transmission of the diseases or ways to control it. According to FAO, the pig population in Mozambique has increased from 170,000 to 1.4 million in 20 years, an eight fold increase, which is most significant in the North [39]. With the increase of the pig population and the presence of risk factors for parasite transmission, the burden of this zoonosis will further exacerbate the life of people and pigs living in the district.

Our study has some limitations. Due to lack of information on frequency and disability weights, only the most frequent clinical symptoms of NCC (epilepsy and headache) were included in the estimates [40]. Headache is a common and non-specific symptom, and while it is a recognized association with NCC it is often not diagnosed as a symptom associated with NCC as such. In this burden assessment headache was only included as an additional symptom among the patients suffering from NCC-associated epilepsy, possibly leading to an under-estimated total burden.

The proportion of the total costs due to hospitalization was attributed to the assumption that patients injured during an epileptic seizure would be hospitalized. Furthermore in Africa the causes of epilepsy are often attributed to supernatural forces that lead to stigmatization, marginalization and exclusion of diseased people [23]. These are social aspects that are difficult to capture however recently the Kilifi Stigma Scale for Epilepsy, a new tool to measure perceived stigma among people with epilepsy was developed and validated [41]. This tool could assess in a study on young people with epilepsy conducted in Tanzania the impact of epilepsy on social transitioning outcomes and showed that these pupils with epilepsy were more likely to experience unfavorable educational, relationship and employment outcomes in the transition to adult life than controls [42]. This calls for models for disease burden estimates to be further improved and include sociological aspects to provide a more accurate and comprehensive estimate of the burden of disease.

In regards to porcine cysticercosis, due to the unavailability of a reliable number of the total pig population, this number had to be estimated. This will lead to inaccuracy in the derived prevalence and thus number of infected pigs and pig production losses. Furthermore, with the lack of organized/official market for pigs and associated listing, the value of a pig slaughtered/sold will remain a qualified estimate. The same regards the per cent loss in value due to cysticercosis – a reduced price may change the decision of a farmer to slaughter or sell.

It should be underlined that the epidemiological parameters used to estimate the health and economic burden in this study were collected in parallel from humans and pigs in a specific district in northern Mozambique, which is different from other studies.

From the results of this study and the comparison with the studies carried out previously on the burden of *T. solium* it is important to underline that both, the health and economic estimations depend on prevalence estimates, therefore it is essential to have accurate data in order to generate accurate burden estimates.

## Conclusion

Results of this study showed that in Angónia district, Tete province, Mozambique endemic to *T. solium* the infection affects the livelihood of the subsistence farmers by reducing their societal and economic wellbeing. A large number of individuals suffer from NCC-associated epilepsy and headache. Healthy years of life are lost and people are left living with disabilities. Infected pork poses a serious risk to the community and hinders the economy of smallholder farmers. Cost for treatment and hospitalization of patients with NCC-associated epilepsy, and lack of productivity of suffering patients further lead to an important obstacle to socioeconomic development.

Feasible solutions framed within a country specific algorithm and stepwise approaches are needed to control the parasite in the country.
